# Association of vitreous CREG1 with diabetic macular edema response and edema resolution following ranibizumab therapy

**DOI:** 10.3389/fmed.2026.1804072

**Published:** 2026-04-30

**Authors:** Linling Zhu, Dongmei Ding, Haixiang Xiao, Ying Cao, Danni Li, Gang Cao

**Affiliations:** 1 Lixiang Eye Hospital of Soochow University, Suzhou, Jiangsu, China; 2 Department of Ophthalmology, Huizhou Hospital of Traditional Chinese Medicine, Huizhou, Guangzhou, China

**Keywords:** anti-VEGF therapy, cellular repressor of E1A-stimulated genes 1, diabetic macular edema, diabetic retinopathy, ranibizumab

## Abstract

**Background:**

This study examined the association between vitreous cellular repressor of E1A-stimulated genes 1 (CREG1) levels and the therapeutic response to ranibizumab in patients with diabetic macular edema (DME).

**Methods:**

In this retrospective study, 189 DME patients receiving ranibizumab were categorized into responders (*n* = 158) and non-responders (*n* = 31) based on edema resolution. Clinical variables and vitreous CREG1 levels were analyzed. LASSO regression and multivariate logistic models identified factors influencing treatment response. Patients were followed for 12 months, with central macular thickness (CMT), retinal nerve fiber layer (RNFL) thickness, and best-corrected visual acuity (BCVA) compared between groups and correlated with CREG1.

**Results:**

Non-responders had a higher prevalence of serous retinal detachment and elevated HbA1c, neutrophil-to-lymphocyte ratio (NLR), and platelet-to-lymphocyte ratio (PLR), but lower CREG1 and estimated glomerular filtration rate (eGFR) (all *P* < 0.05). LASSO-selected factors (CREG1, OCT classification, HbA1c, NLR, PLR, eGFR) were analyzed by logistic regression: OCT class, HbA1c, NLR, and PLR were risk factors for non-response, whereas CREG1 and eGFR were protective factors (all *P* < 0.05). Throughout follow-up, non-responders exhibited worse BCVA, greater CMT, and thicker RNFL (*P* < 0.05). Vitreous CREG1 was negatively correlated with CMT, RNFL, and BCVA at all timepoints (all *P* < 0.05).

**Conclusion:**

Lower vitreous CREG1 is associated with poor response to ranibizumab in DME. CREG1, along with OCT features, HbA1c, NLR, PLR, and eGFR, significantly influences treatment outcomes and correlates with long-term anatomical and visual prognosis.

## 1 Introduction

Diabetic retinopathy (DR), as one of the most common microvascular complications of diabetes, has become a significant global public health challenge due to the vision impairment it causes (1–3). According to Hirano et al. (4), approximately 30% of patients with DR develop secondary diabetic macular edema (DME), which is pathologically characterized by retinal thickening in the macular area, fluid accumulation, and disruption of the blood-retinal barrier, ultimately leading to irreversible vision loss. Although anti-vascular endothelial growth factor (VEGF) agents, which inhibit neovascularization and reduce vascular permeability, have become the first-line treatment for DME, clinical practice shows that approximately 20–40% of patients exhibit poor or short-lived responses to anti-VEGF therapy, suggesting that the pathogenesis of diabetic macular edema (DME) involves a complex network of molecular regulation, highlighting the urgent need to explore new biomarkers and therapeutic targets (5, 6).

Recent studies have found that mechanisms involving inflammatory factors play a synergistic role in the development and progression of DME (7). For instance, high levels of inflammatory mediators in the vitreous microenvironment can activate glial cells and Müller cells, further aggravating the disruption of the blood-retinal barrier (8). In addition, morphological markers on optical coherence tomography (OCT), such as intraretinal cyst formation and disorganization of the outer plexiform layer, have been confirmed to be associated with the prognosis of anti-VEGF therapy, yet there is still a lack of molecular markers that can predict treatment response at an early stage. Against this backdrop, researchers have gradually turned their attention to regulatory proteins within the vitreous cavity related to cellular homeostasis and tissue repair (9). Cellular repressor of E1A-stimulated genes 1 (CREG1), as a novel regulator of cellular homeostasis, plays a critical role in maintaining extracellular matrix balance and suppressing inflammatory responses; however, its expression characteristics in DME and its association with anti-VEGF treatment response remain unclear (10, 11). Currently, although standardized anti-VEGF treatment regimens can significantly improve macular edema and visual prognosis in most patients, the economic burden and potential complications associated with repeated intravitreal injections still limit its long-term efficacy. Studies have shown that the early degree of edema resolution following anti-VEGF therapy is closely related to baseline levels of vitreous inflammatory factors, suggesting that the molecular characteristics of the vitreous microenvironment may influence treatment response (12). Therefore, elucidating the key molecular mechanisms regulating DME progression and treatment response is of great significance for optimizing stratified treatment strategies and reducing ineffective medical interventions, yet there are currently few clinical studies in this area.

Based on this, the present study focuses on the potential role of vitreous CREG1 protein in ranibizumab therapy for DME. By analyzing the expression levels of CREG1 in vitreous samples from patients with DME, this study aims to clarify whether CREG1 and related clinical indicators can serve as biomarkers for predicting the efficacy of ranibizumab therapy or as potential therapeutic targets. The goal is to provide new evidence for molecular classification in patients with DME and to open new avenues for the development of individualized treatment strategies.

## 2 Materials and methods

### 2.1 Study subjects

A total of 189 patients with DME admitted between February 2024 and October 2025 were retrospectively included. All patients received ranibizumab therapy in our hospital, and treatment responses were recorded. According to the degree of edema resolution, patients were divided into an effective response group and a non-response group. The study was approved by the hospital ethics committee and complied with the Declaration of Helsinki.

### 2.2 Inclusion and exclusion criteria

Inclusion criteria: (1) Complete clinical data and follow-up information; (2) Meeting the diagnostic criteria for DME in the *Clinical Practice Guidelines for Diabetic Retinopathy* (13); (3) Unilateral onset; (4) Central macular thickness (CMT) > 300 μm; (5) Met the indications for ranibizumab therapy and received treatment in our hospital; (6) Basic communication ability and cooperation to complete examinations.

Exclusion criteria: (1) History of mental illness, cognitive impairment or drug dependence; (2) Previous severe cataracts, eye trauma or other eye diseases; (3) Combined with organic lesions of the heart, liver, kidney and other organs; (4) Combined with blood system diseases; (5) History of previous eye surgery.

### 2.3 General information

The medical records of patients with DME who met the inclusion criteria were collected based on the hospital information management system. The data included age, gender (male/female), body mass index, education level (junior high school and below/high school and above), hypertension (in accordance with the diagnostic criteria of The Japanese Society of Hypertension Guidelines for Self-monitoring of Blood Pressure at Home (Second Edition) (14) (yes/no), smoking history (yes/no), drinking history (yes/no), duration of diabetes, type of diabetes (type 1 diabetes/type 2 diabetes), systolic blood pressure, and diastolic blood pressure.

### 2.4 Grouping method

Patients were classified into responders and non-responders based on the degree of edema resolution and visual improvement observed at the 12-month follow-up visit. According to the Clinical Disease Diagnosis and Efficacy Evaluation Criteria (15), effective response was defined as complete resolution of effusion and/or improvement of visual acuity by more than 5 letters after treatment. Non-response was defined as meeting either of the following criteria: (1) anatomical non-response: < 10% reduction in CMT from baseline with persistent intraretinal fluid (IRF) or subretinal fluid (SRF), or presence of new IRF/SRF; or (2) functional non-response: loss of ≥ 5 ETDRS letters in best-corrected visual acuity (BCVA) from baseline. Patients meeting both criteria were classified as complete non-responders. All patients received ranibizumab injections according to a monthly schedule for the first three months, followed by as-needed injections based on OCT findings. The timing and frequency of injections were recorded throughout the 12-month follow-up to ensure consistency in treatment administration.

### 2.5 Laboratory index testing

On the day of examination, 8 mL of venous blood was collected from each patient and placed into two vacuum tubes. HbA1c was measured using a specific protein analyzer (Beckman Coulter, IMMAGE 800). The other tube was centrifuged at room temperature (LL900 centrifuge, Luoyang Hongshi Machinery Equipment Co., Ltd.) at 3000 r/min for 10 min to separate serum. Interleukin-6 (IL-6) was measured using a Redu RT-500 automatic microplate reader with an ELISA kit (Shenzhen Kerunda Bioengineering Co., Ltd., produced by Japan IBL), following the manufacturer’s instructions. A fully automatic biochemical analyzer (Beckman Coulter, Unicel Dxc 800 Synchro) was used to measure fasting plasma glucose (FPG) and serum creatinine (Scr), and the eGFR was calculated using eGFR = 186 × (Scr/88.4)−1.154 × (age)−0.203 (female × 0.742). A CEM Premier3000 blood gas analyzer (Instrumentation Laboratory, United States) was used to measure absolute neutrophil count, absolute lymphocyte count, and platelet count, and the neutrophil-to-lymphocyte ratio (NLR) and platelet-to-lymphocyte ratio (PLR) were calculated.

### 2.6 Optical coherence tomography (OCT) indicators

A Heidelberg OCT device (Germany) was used for rapid scanning with 31 horizontal lines to scan both eyes of the patients. Pre-treatment OCT classification and post-treatment CMT, retinal nerve fiber layer thickness (RNFL), and best-corrected visual acuity letter scores (BCVA) at 3, 6, and 12 months were collected. According to morphology, patients were classified into three types: serous retinal detachment (SRD), cystoid macular edema (CME), and diffuse retinal thickening (DRT). DRT is characterized by sponge-like swelling in the macular region with uniformly reduced intraretinal reflectivity. CME is characterized by low-reflectivity cystoid spaces in the macular region separated by high-reflectivity septa. SRD is characterized by elevation of the macular region with separation of the retinal pigment epithelium layer, showing transparent low-reflectivity cavities between them.

### 2.7 Vitreous CREG1 measurement

Vitreous fluid (0.1–0.2 mL) was collected via routine pars plana puncture under sterile conditions prior to the first intravitreal ranibizumab injection. All samples were obtained using a standardized collection procedure to ensure consistency across patients and to avoid contamination with blood or retinal tissue. Immediately after collection, the samples were aliquoted into pre-chilled heparin sodium anticoagulant tubes and stored at −80°C to avoid repeated freeze-thaw cycles. Hyaluronidase was added to a final concentration of 200 U/mL and incubated at 37°C for 30 min. An equal volume of PBS buffer containing 1% BSA was added, mixed, and centrifuged at 4000 rpm at 4°C for 20 min. The supernatant was filtered through a 0.22 μm membrane to remove insoluble particles. According to the ELISA kit instructions (Shanghai Zeye Biotechnology Co., Ltd.), reagents were prepared. Standards were prepared using artificial vitreous fluid, and sample diluent contained 1% BSA. 50 μL of the sample and 50 μL of detection antibody were added and incubated at 37°C for 2.5 h with shaking at 500 rpm. After discarding the liquid, 300 μL of enhanced washing solution was added to each well, washed three times, and dried. A 100 μL of substrate solution (TMB) was added and incubated at 37°C in the dark for 15 min. Then, 50 μL of stop solution (2M H_2_SO_4_) was added, mixed, and detection was completed within 10 min. After the reaction was stopped, dual-wavelength detection was performed using a microplate reader with a main wavelength of 450 nm and a reference wavelength of 630 nm. A standard curve was plotted based on the absorbance and concentration of the standards, and the CREG1 concentration in the samples was calculated accordingly.

### 2.8 Statistical analysis

SPSS 26.0 and R 4.3.2 software were used for statistical analysis and graphical plotting. For measurement data with a normal distribution, independent-samples *t*-test was used, expressed as ($\bar{x}$ ± s); for non-normally distributed data, the Mann-Whitney U test was used, expressed as median and interquartile range [M (P_25_, P_75_)]. Categorical data were analyzed using the *x*^2^ test, expressed as frequency, with *P* < 0.05 indicating statistical significance. Pearson correlation analysis was used to analyze the relationship between differential indicators and the response of DME, as well as the association between CREG1 and long-term treatment outcomes. To reduce the impact of multicollinearity on regression results and avoid overfitting, the factors screened were analyzed using the “glmnet” package in R based on the least absolute shrinkage and selection operator (LASSO) regression, and the final screened variables were included in multivariate logistic regression to identify independent risk factors influencing the response of DME.

## 3 Results

### 3.1 Comparison of general clinical data between the non-response group and the effective response group

Among the 189 patients with DME who received ranibizumab treatment, the incidence of effective response was 83.60%, and these patients were classified into the effective response group (*n* = 158). The incidence of non-response was 16.40%, and these patients were classified into the non-response group (*n* = 31). The proportion of SRD (13/31 vs. 25/158) in the non-response group was higher than that in the effective response group, and the difference was statistically significant (*P* < 0.05). However, there were no statistically significant differences between the non-response group and the effective response group in terms of age, gender, body mass index, education level, presence of hypertension, smoking history, drinking history, duration of diabetes, type of diabetes, systolic blood pressure, and diastolic blood pressure (*P* > 0.05), as shown in Table 1.

**TABLE 1:** Comparison of general clinical data between the non-response group and the effective-response group.

Indicator		Non-response group (*n* = 31)	Effective response group (*n* = 158)	*χ^2^* / *t*	*P*
Age (years)		60.78 ± 6.48	60.55 ± 6.70	0.176	0.861
Gender (n)	Male	18	88	0.059	0.808
	Female	13	70		
Body Mass Index (kg/m^2^)		23.78 ± 1.36	23.69 ± 1.39	0.331	0.741
Education Level (n)	Junior high school and below	20	88	0.823	0.364
	High school and above	11	70		
Combined hypertension (n)	Yes	3	19	0.139	0.709
	No	28	139		
Smoking history (n)	Yes	10	45	0.179	0.672
	No	21	113		
Drinking history (n)	Yes	12	44	1.466	0.226
	No	19	114		
Diabetes duration (year)		7.74 ± 1.45	7.49 ± 1.33	0.943	0.347
Types of diabetes (n)	Type 1 diabetes	6	13	3.548	0.060
	Type 2 diabetes	25	145		
Systolic Blood Pressure (mmHg)		132.79 ± 13.77	128.09 ± 17.46	1.414	0.159
Diastolic Blood Pressure (mmHg)		80.12 ± 9.55	79.12 ± 10.01	0.512	0.609
OCT Classification (n)	SRD	13	25	11.067	0.004
	CME	8	64		
	DRT	10	69		

OCT, optical coherence tomography; SRD, serous retinal detachment, CME; cystoid macular edema, DRT, diffuse retinal thickening.

### 3.2 Comparison of laboratory indicators between the non-response group and the effective response group

To illustrate the laboratory indicators between the non-response group and the effective response group, the pre-treatment laboratory parameters were compared between the two groups. The results showed that there were no statistically significant differences in IL-6, fasting plasma glucose (FPG), and serum creatinine between the non-response and effective response groups (*P* > 0.05). However, the non-response group had higher levels of HbA1c (7.83 ± 1.51 vs. 7.04 ± 1.30%), neutrophil-to-lymphocyte ratio (NLR) (2.09 ± 0.86 vs. 1.68 ± 0.46), and platelet-to-lymphocyte ratio (PLR) (118.03 ± 37.35 vs. 92.55 ± 25.30) compared to the effective response group, while CREG1 (48.95 ± 5.37 vs. 63.56 ± 8.29 pg/mL) and estimated glomerular filtration rate (eGFR) (19.67 ± 6.15 vs. 24.89 ± 8.19 mL/min/1.73 m^2^) were significantly lower in the non-response group than in the effective response group (*P* < 0.05). Details are shown in Table 2.

**TABLE 2:** Comparison of laboratory indicators between the non-response group and the effective response group.

Indicator	Non-response group (*n* = 31)	Effective response group (*n* = 158)	*t*	*P*
CREG1 (pg/mL)	48.95 ± 5.37	63.56 ± 8.29	9.099	<0.001
IL-6 (pg/mL)	23.15 ± 3.58	22.38 ± 3.04	1.251	0.212
NLR	2.09 ± 0.86	1.68 ± 0.46	3.834	<0.001
PLR	118.03 ± 37.35	92.55 ± 25.30	4.701	<0.001
FPG (mmol/L)	10.12 ± 3.33	10.36 ± 3.45	0.356	0.722
Serum creatinine (mg/dL)	3.55 ± 0.98	3.31 ± 1.21	1.039	0.300
eGFR (mL/min/1.73 m^2^)	19.67 ± 6.15	24.89 ± 8.19	3.364	0.001
HbAlc (%)	7.83 ± 1.51	7.04 ± 1.30	3.010	0.003

HbAlc, glycated hemoglobin; IL-6, interleukin-6; NLR, neutrophil-to-lymphocyte ratio; PLR, platelet-to-lymphocyte ratio; FPG, fasting plasma glucose; Scr, serum creatinine; eGFR, estimated glomerular filtration rate, CREG1, cellular repressor of E1A, stimulated genes 1.

### 3.3 Correlation between CREG1, OCT classification, HbA1c, NLR, PLR, eGFR and the response of DME

**FIGURE 1:**
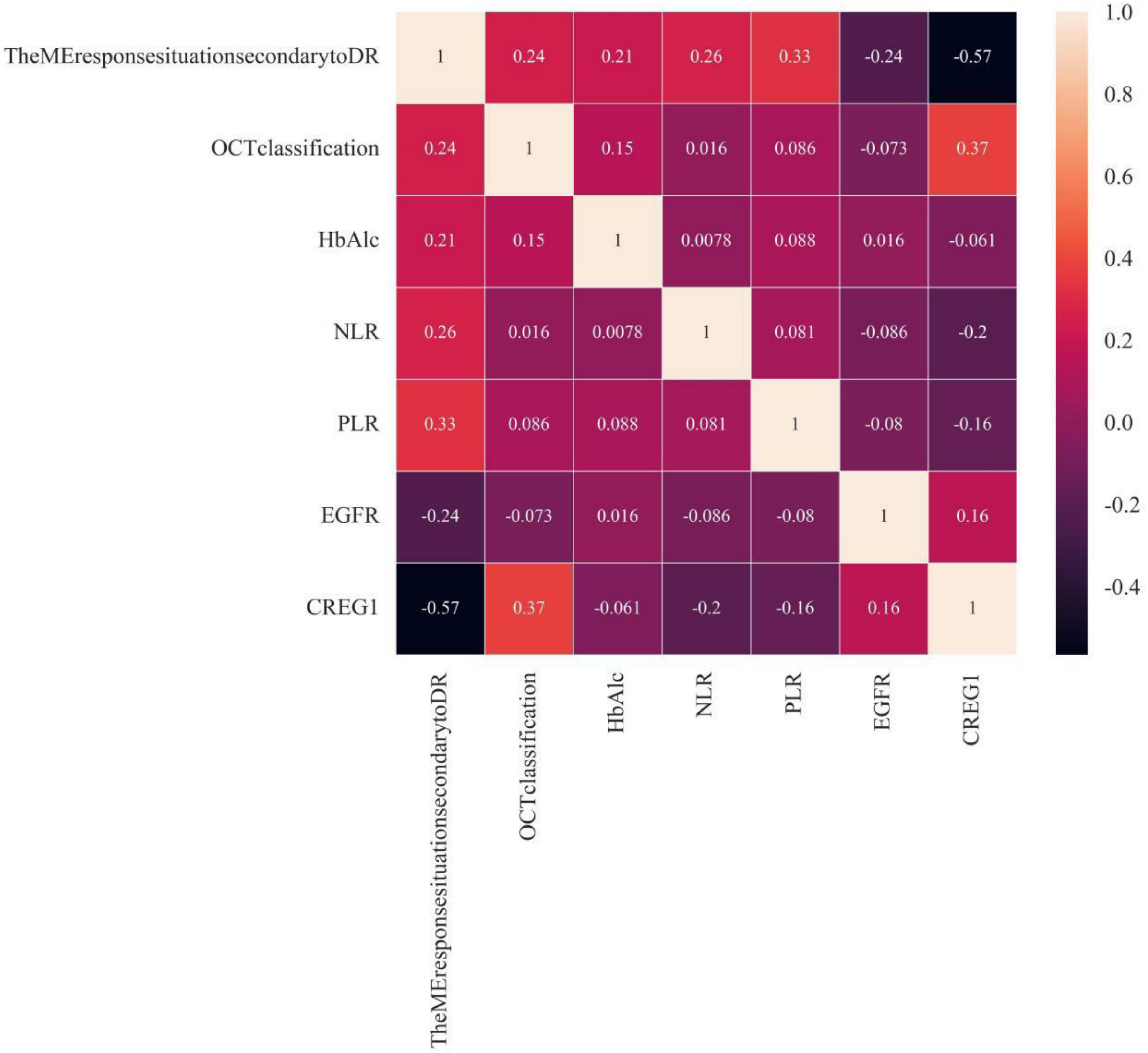
Correlation between CREG1, OCT classification, HbA1c, NLR, PLR, eGFR and the response of DME.

Pearson correlation analysis was conducted to assess the correlation between OCT classification, HbA1c, NLR, PLR, eGFR, and the response of DME. The results showed that OCT classification, HbA1c, NLR, and PLR were positively correlated with the response of DME (*r* = 0.24, 0.21, 0.26, 0.33, *P* < 0.05), while CREG1 and eGFR were negatively correlated with the response of DME (*r* = −0.57, −0.24, *P* < 0.05). These findings indicate that there is correlation between CREG1, OCT classification, HbA1c, NLR, PLR, eGFR, and the response of DME, and the correlation with CREG1 is significant, as shown in Figure 1.

### 3.4 LASSO regression analysis between the non-response group and effective response group

**FIGURE 2:**
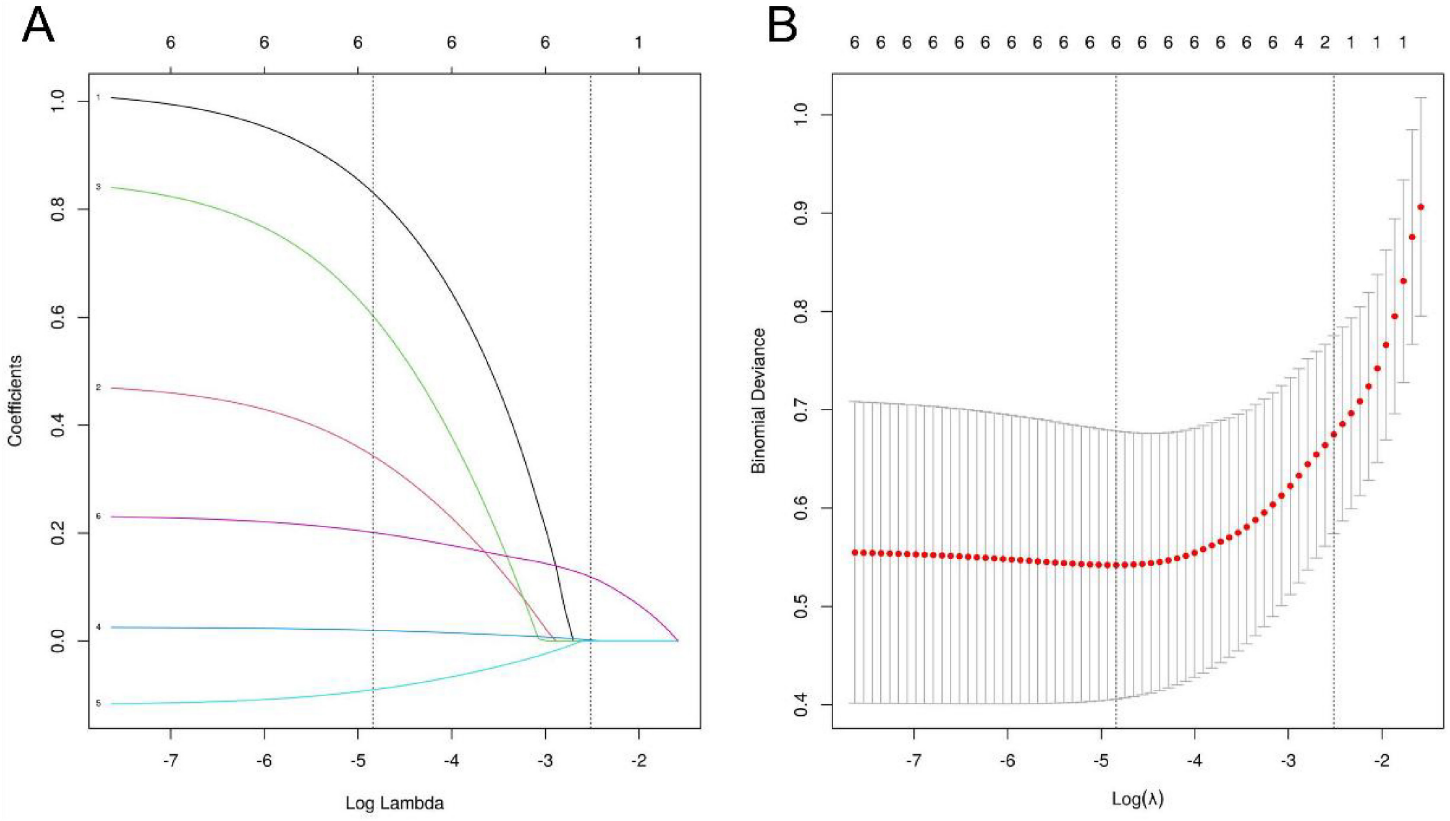
LASSO regression analysis. The lower horizontal axis of **(A)** represents the size of the log (λ) value in the lasso regression model, the upper horizontal axis represents the number of non-zero coefficient variables in the corresponding model at this time, and the vertical axis represents the coefficient value of each feature in the model. Each curve shows the change trajectory of the regression coefficient of each independent variable. The vertical axis of **(B)** is the mean square error, the red vertical line is lambda.min, which is the number of independent variables in the model corresponding to the minimum mean square error, and the black vertical line lambda.lse is the number of independent variables when the distance from the minimum mean square error is one standard error.

To reduce multicollinearity and avoid overfitting, a LASSO regression model was used for variable selection, screening potential influencing factors with statistical significance. Ten-fold cross-validation was applied to determine the optimal λ value. As the penalty coefficient λ increased, the coefficients of the independent variables were gradually compressed (Figure 2A). The λ value corresponding to the minimum error in the ten-fold cross-validation (λ = 0.008) was selected as the optimal value (Figure 2B), and six meaningful factors were identified: CREG1, OCT classification, HbA1c, NLR, PLR, and eGFR.

### 3.5 Logistic regression analysis of the response to ranibizumab therapy in DME

Variables identified through univariate analysis and LASSO regression analysis were included in a multivariate logistic regression analysis. The results indicated that OCT classification, HbA1c, NLR, and PLR were risk factors for the response of DME (OR = 1.822, 1.503, 2.946, 1.027, *P* < 0.05), while CREG1 and eGFR were protective factors for the response of DME (OR = 0.791, 0.923, *P* < 0.05). These findings suggest that CREG1, OCT classification, HbA1c, NLR, PLR, eGFR, and are influencing factors for the response of DME. Details are presented in Table 3.

**TABLE 3:** Logistic regression analysis of the response to ranibizumab therapy in DME.

Factor	Regression coefficient	Standard error	Wald χ^2^	*P*-value	OR	OR 95% CI
CREG1	−0.234	0.041	32.308	<0.001	0.791	0.730∼0.858
OCT type	0.600	0.219	7.527	0.006	1.822	1.187∼2.796
HbAlc	0.407	0.165	6.130	0.013	1.503	1.089∼2.075
NLR	1.080	0.376	8.270	0.004	2.946	1.411∼6.151
PLR	0.026	0.007	13.318	<0.001	1.027	1.012∼1.041
eGFR	−0.081	0.038	4.537	0.033	0.923	0.857∼0.994

HbAlc, glycated hemoglobin; NLR, neutrophil-to-lymphocyte ratio; PLR, platelet-to-lymphocyte ratio; eGFR, glomerular filtration rate; CREG1, E1A-activated gene repressor 1; OCT, optical coherence tomography.

### 3.6 ROC analysis of six single indicators

**FIGURE 3:**
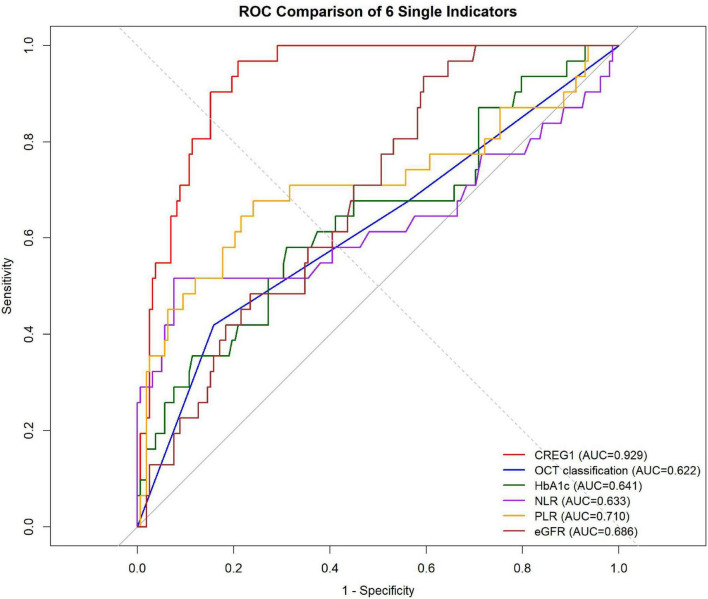
ROC analysis of 6 single indicators for predicting non-response to ranibizumab therapy in patients with DME. ROC, receiver operating characteristic; AUC, area under the curve; OCT, optical coherence tomography; HbA1c, glycated hemoglobin; NLR, neutrophil-to-lymphocyte ratio; PLR, platelet-to-lymphocyte ratio; eGFR, estimated glomerular filtration rate.

Receiver operating characteristic (ROC) curve analysis was performed to evaluate the predictive performance of the six single indicators for non-response to ranibizumab therapy in patients with DME. As shown in Figure 3, vitreous CREG1 exhibited the highest discriminative ability, with an AUC of 0.929. Among the remaining indicators, PLR showed moderate predictive performance (AUC = 0.710), followed by eGFR (AUC = 0.686), HbA1c (AUC = 0.641), NLR (AUC = 0.633), and OCT classification (AUC = 0.622). These findings indicate that vitreous CREG1 outperformed the other single indicators in predicting treatment non-response and may represent the most informative individual biomarker for identifying patients at high risk of poor response to ranibizumab therapy.

### 3.7 Comparison of Long-term visual recovery and retinal nerve fiber layer thickness between the non-response and effective response groups

At 3, 6, and 12 months after treatment, the CMT levels in the non-responder group (475.22 ± 55.37 vs. 444.46 ± 46.39; 410.37 ± 50.26 vs. 377.45 ± 41.59 μm; 386.67 ± 84.11 vs. 311.01 ± 85.16 μm, respectively) were higher than those in the responder group. Similarly, RNFL levels (115.06 ± 6.32 vs. 111.34 ± 6.02 μm; 110.47 ± 5.49 vs. 106.35 ± 5.89 μm; 105.46 ± 5.13 vs. 99.87 ± 5.36 μm, respectively) and BCVA (1.09 ± 0.15 vs. 0.98 ± 0.21 logMAR; 0.92 ± 0.11 vs. 0.82 ± 0.10 logMAR; 0.84 ± 0.14 vs. 0.71 ± 0.09 logMAR, respectively) in the non-responder group were higher than those in the responder group, with statistically significant differences (*P* < 0.05). Details are presented in Table 4.

**TABLE 4:** Comparison of long-term visual recovery and retinal nerve fiber layer thickness between the non-response and effective response groups.

Indicator		Non-response group (*n* = 31)	Effective response group (*n* = 158)	*t*	*P*
CMT (μm)	3 months after treatment	475.22 ± 55.37	444.46 ± 46.39	3.266	0.001
	6 months after treatment	410.37 ± 50.26	377.45 ± 41.59	3.888	<0.001
	12 months after treatment	386.67 ± 84.11	311.01 ± 85.16	4.532	<0.001
RNFL (μm)	3 months after treatment	115.06 ± 6.32	111.34 ± 6.02	3.120	0.002
	6 months after treatment	110.47 ± 5.49	106.35 ± 5.89	3.599	<0.001
	12 months after treatment	105.46 ± 5.13	99.87 ± 5.36	5.355	<0.001
BCVA (logMAR)	3 months after treatment	1.09 ± 0.15	0.98 ± 0.21	2.778	0.006
	6 months after treatment	0.92 ± 0.11	0.82 ± 0.10	5.007	<0.001
	12 months after treatment	0.84 ± 0.14	0.71 ± 0.09	6.636	<0.001

CMT, the central macula thickness; RNFL, the retinal nerve fiber layer thickness; BCVA, the best corrected visual acuity in letters *P<0.05, **P<0.01.

### 3.8 Correlation between CREG1 and long-term visual recovery and retinal nerve layer thickness

**FIGURE 4:**
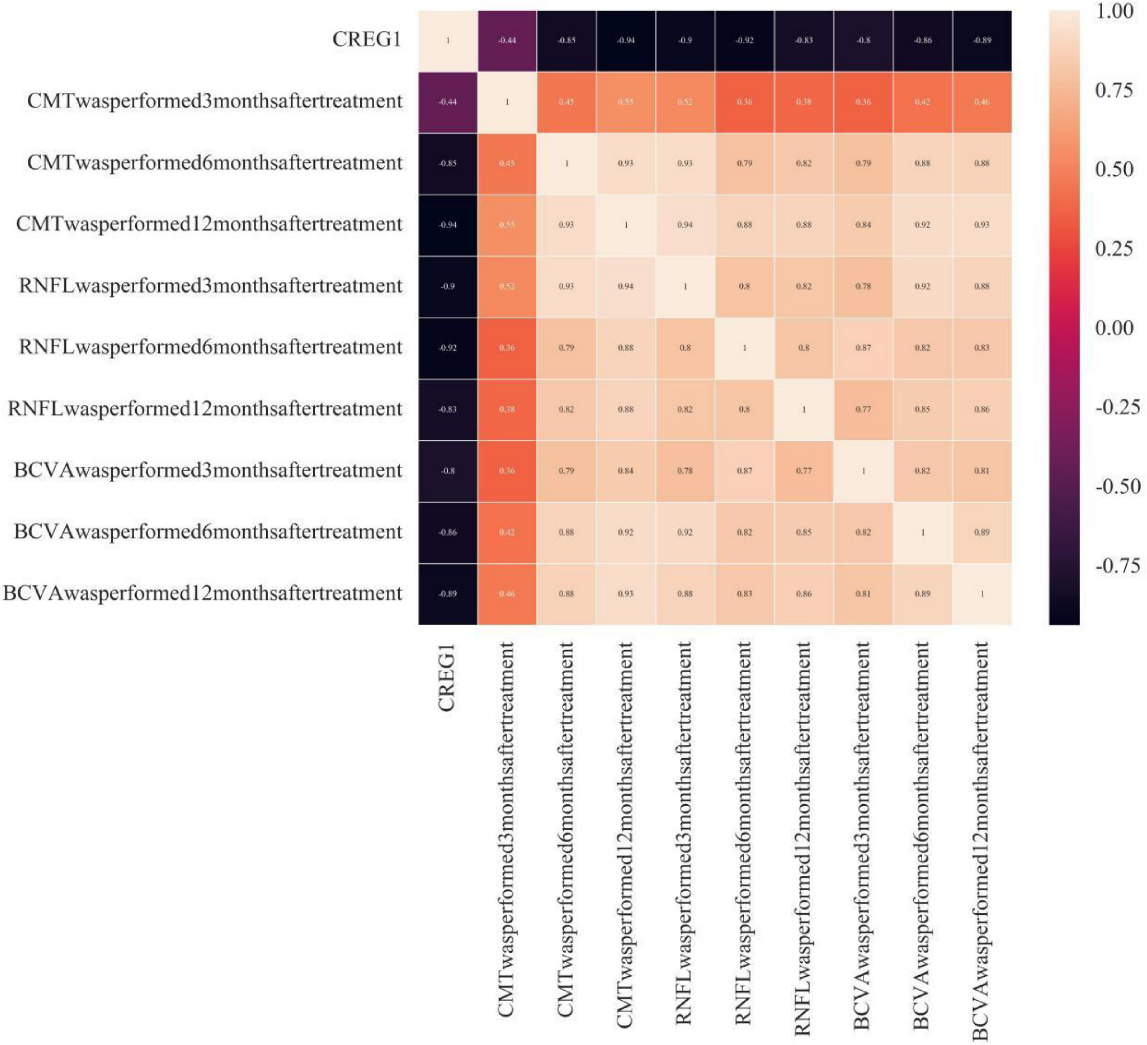
Correlation between CREG1 and long-term visual recovery and retinal nerve layer thickness.

Pearson correlation analysis was used to assess the correlation between CREG1 and long-term visual recovery and retinal nerve fiber layer thickness. The results showed that CREG1 was negatively correlated with RNFL, CMT, and BCVA at 3, 6, and 12 months after treatment (*r* = −0.441, −0.854, −0.940, −0.896, −0.919, −0.834, −0.799, −0.861, −0.888; *P* < 0.05). These findings indicate a significant correlation between CREG1 and long-term visual recovery and retinal nerve fiber layer thickness (see Figure 4).

## 4 Discussion

DME is the most common cause of vision loss among ophthalmic patients in developed countries. Although the precise mechanisms underlying its development have not been fully elucidated, VEGF-mediated disruption of the blood-retinal barrier, which subsequently triggers a series of inflammatory responses, is a widely recognized cause and mechanism of the disease (16, 17). Currently, anti-VEGF therapy is commonly employed in clinical practice, effectively preventing neovascularization, reducing VEGF-induced damage to the blood-retinal barrier, delaying and preventing the progression of macular edema, reducing the degree of tissue edema, and improving patients’ visual function (18). However, studies have found that clinical outcomes are not ideal in some patients, and individual differences may lead to variable therapeutic efficacy of anti-VEGF treatment (19). In this study, the effective response rate of 189 patients with DME following ranibizumab treatment was 83.60% (*n* = 158, effective response group), while the non-response rate was 16.40% (*n* = 31, non-response group), consistent with previous clinical studies, confirming that treatment remains ineffective in a subset of patients. Early identification of influencing factors can provide reference for targeted nursing measures.

Previous studies have confirmed that CREG1 is an important regulatory factor for the maturation and differentiation of vascular smooth muscle cells, expressed early during embryonic vascular development and involved in the regulation of angiogenesis (20). In this study, the CREG1 levels in the non-response group were lower than those in the effective response group and were negatively correlated with the treatment response, indicating that low CREG1 expression may contribute to the non-response to ranibizumab therapy in patients with DME. The potential mechanism may be that CREG1 activates the PI3K/AKT signaling pathway, enhances the expression of endothelial junction proteins, and reduces vascular leakage. Under low CREG1 levels, endothelial barrier function is impaired, making it difficult to control vascular leakage even when anti-VEGF drugs inhibit VEGF-A, and reducing pro-apoptotic factors, thereby maintaining endothelial cell survival and preventing abnormal neovascularization following vascular degradation, leading to treatment resistance. Previous studies have shown that CREG1 can inhibit the activity of pro-inflammatory transcription factors, reduce the release of inflammatory mediators, while hyperglycemia-induced oxidative stress and chronic inflammation can suppress CREG1 expression, leading to a persistent pro-inflammatory microenvironment that weakens the anti-VEGF effect on vascular leakage. Furthermore, CREG1 can promote the differentiation of macrophages toward an anti-inflammatory phenotype through STAT3 signaling, reducing pro-inflammatory macrophage infiltration and suppressing inflammation-driven blood-retinal barrier damage (21). Data also indicate that CREG1 is closely related to VEGF signaling pathways and oxidative stress/fibrosis (22, 23). On one hand, CREG1 reduces the transcriptional activity of VEGFR-2 through epigenetic modification, attenuating VEGF-A signaling. Low CREG1 expression can lead to VEGFR-2 overactivation, counteracting the efficacy of anti-VEGF drugs. On the other hand, CREG1 activates the Nrf2 pathway and inhibits the TGF-β/Smad3 pathway, upregulating antioxidant enzymes such as superoxide dismutase and glutathione peroxidase, reducing extracellular matrix deposition, alleviating hyperglycemia-induced oxidative damage, delaying fibrosis and scar formation in the macular region, and improving drug penetration and efficacy. Conversely, low CREG1 expression can weaken these protective mechanisms and reduce treatment response.

This study also found that CREG1 was negatively correlated with RNFL, CMT and BCVA (*P* < 0.05). It is speculated that CREG1, as a protein involved in cell differentiation and inflammation regulation, plays multiple roles in the pathological process of DME. The essential pathology in these patients involves hyperglycemia-induced VEGF upregulation, leading to blood-retinal barrier breakdown and fluid accumulation in the macular area, increasing CMT. RNFL thickening reflects acute retinal nerve fiber layer edema or inflammatory infiltration, related to persistent inflammation activating glial cell proliferation or axonal swelling. BCVA reduction is directly associated with increased CMT and abnormal RNFL; edema compresses photoreceptor cells and disrupts retinal layer structure, impairing visual signal transmission. Non-responders may exhibit “inflammatory escape,” where VEGF inhibition fails to block non-VEGF pathways, leading to persistent vascular permeability, elevated CMT and RNFL over the long term, vascular dysregulation, retinal ischemia, and neuronal damage, worsening BCVA. CREG1 may activate pro-inflammatory pathways such as NF-κB, inhibit axonal transport, stimulate glial proliferation and inflammatory exudation, causing axonal swelling and RNFL thickening. CREG1’s anti-apoptotic properties may prolong inflammatory cell survival, increase vascular endothelial permeability, sustain local inflammation, and promote plasma leakage into the macula, increasing CMT. Persistently high CREG1 levels may also impair retinal ganglion cell (RGC) function, reducing visual signal input to the brain, leading to BCVA decline. In summary, as a novel regulatory protein maintaining vascular homeostasis, CREG1 can synergistically enhance anti-VEGF treatment efficacy in DME by inhibiting inflammation, stabilizing the vascular barrier, modulating VEGF signaling, and reducing oxidative stress/fibrosis. Low CREG1 expression may lead to the dysregulation of multiple pathological mechanisms, ultimately causing treatment resistance, but current clinical research on this is limited and requires further investigation.

HbA1c refers to the product formed by the non-enzymatic binding of blood glucose to hemoglobin in red blood cells, while OCT classification refers to the categorization and diagnosis of lesions or tissues based on imaging features obtained by optical coherence tomography, providing high-resolution structural and optical imaging simultaneously (24, 25). This study found that the proportion of SRD and HbA1c levels were higher in the non-response group, and OCT classification and HbA1c were positively correlated with treatment response, both being risk factors for non-response to ranibizumab therapy in DME. It is hypothesized that elevated HbA1c, which has a higher oxygen affinity, can exacerbate retinal hypoxia, promote persistent capillary dilation, and increase macular retinal thickness. Clinical studies have indicated that HbA1c is closely related to ME regression, with higher levels hindering edema resolution and increasing treatment difficulty and the risk of non-response (26). OCT classification divides patients into DRT, CME, and SRD types, with DRT commonly seen in the outer plexiform layer, SRD located between the RPE and the photoreceptor outer segment, and CME in the outer plexiform and inner nuclear layers (27). Our data showed that the proportion of SRD was higher in the non-response group, consistent with findings by Hui et al. (28). This may be because DRT and CME are mainly caused by inner blood-retinal barrier disruption, related to VEGF, while SRD is associated with inflammatory factors and outer barrier dysfunction leading to subretinal fluid accumulation. Anti-VEGF drugs effectively reduce leakage in DRT and CME types but are less effective in SRD. NLR reflects systemic inflammation and immune balance; PLR comprehensively reflects coagulation, inflammation, and immune status; eGFR is a core indicator of renal function, reflecting glomerular filtration capacity (29, 30). In this study, NLR and PLR were higher, and eGFR was lower in the non-response group, with these factors being risk factors for non-response. Elevated NLR and PLR indicate systemic inflammation and hypercoagulability, promoting alternative angiogenesis, enhancing endothelial survival, and reducing immune surveillance against angiogenesis, thereby weakening immune regulation and enabling persistent abnormal vascular proliferation. Zhou et al. (31) reported that platelets secrete PDGF, and high NLR and PLR expression indicate abnormal inflammation and hypercoagulation, activating PI3K/Akt and PAR pathways, enhancing endothelial survival and proliferation, and promoting microthrombosis and local thrombin release, which can resist anti-VEGF-induced vascular regression, leading to persistent vascular leakage and edema, increasing non-response risk. Low eGFR suggests pericyte apoptosis, increased VEGF sensitivity, impaired RPE migration and repair, and structural disorder in new vessels, resulting in ineffective blood-retinal barrier reconstruction post-disruption and persistent edema, reducing anti-VEGF efficacy (32). Thus, NLR, PLR, and eGFR may promote non-response through bypass angiogenesis activation, enhanced endothelial survival, and vascular structural defects.

We acknowledge that the biological rationale for CREG1 as a predictive biomarker in DME requires stronger a priori evidence. While CREG1 regulates vascular homeostasis, inflammation, and oxidative stress in other systems (10, 11), its direct involvement in DME pathophysiology remains understudied compared to established mediators such as IL-6 or complement factors. Our selection was based on CREG1’s pleiotropic effects on endothelial barrier function and VEGF signaling (22, 23), yet this foundation is preliminary. In the present study, vitreous samples were collected immediately before intravitreal ranibizumab therapy using a standardized pars plana puncture procedure, which helped ensure consistency in baseline sample acquisition across patients. However, vitreous sampling remains an invasive procedure and may limit the broader clinical applicability of this biomarker in routine practice. In addition, although ROC analysis showed that CREG1 had superior predictive performance as a single indicator, our findings still demonstrate association rather than causation. Low CREG1 expression may reflect a more severe inflammatory or metabolic state rather than directly driving resistance to anti-VEGF therapy. Therefore, the mechanistic interpretations proposed in the present study should be considered preliminary and hypothesis-generating. Further validation in larger prospective cohorts, together with complementary mechanistic studies, is still required before concluding that low vitreous CREG1 independently predicts treatment failure.

However, there are still some shortcomings. (1) Follow-up duration: Anti-VEGF treatment requires long-term follow-up ( > 1 year) to evaluate recurrence and visual maintenance, while our short-term observation may not fully reflect CREG1’s predictive value. (2) Sample size: The limited non-responder sample (*n* = 31) may affect statistical stability. (3) Agent specificity: This study focused exclusively on ranibizumab; whether CREG1 predicts responses to other anti-VEGF agents (aflibercept, bevacizumab, conbercept) requires validation given their distinct molecular targets. (4) Mechanistic validation: The correlational design precludes causal conclusions; animal models (e.g., CREG1 knockout diabetic mice) and in vitro studies are needed to establish whether CREG1 directly modulates blood-retinal barrier integrity. (5) CREG1-eGFR interaction: Renal insufficiency may affect CREG1 clearance or synthesis, potentially confounding observed associations; future studies should examine their pharmacokinetic relationship. (6) Non-invasive application: Although vitreous samples were collected before intravitreal ranibizumab injection using a standardized pars plana puncture procedure, this approach remains invasive and may limit its widespread clinical application. Therefore, prospective validation of serum- or aqueous-based CREG1 models, potentially combined with OCT-based algorithms, is still warranted. (7) CREG1 validation as an outcome indicator: Given its correlation with disease severity, future research is necessary to explore directions such as longitudinal trials assessing CREG1 as a surrogate endpoint for long-term visual outcomes, ensuring robust evidence for its clinical adoption.

Additionally, as the study population is primarily from China, ethnic or regional factors, such as genetic variations in VEGF pathways common in Asian populations, may limit generalizability to other demographics and require multi-ethnic validation.

## 5 Conclusion

In conclusion, OCT classification, HbA1c, NLR, PLR, eGFR, and CREG1 were closely associated with non-response to ranibizumab treatment in patients with DME. Among them, OCT classification, HbA1c, NLR, and PLR were independent risk factors, whereas CREG1 and eGFR were protective factors. Additionally, CREG1 levels were significantly correlated with long-term visual recovery and retinal nerve fiber layer thickness. Nevertheless, given the limited representativeness of the study cohort and the fact that the incremental predictive value of CREG1 beyond conventional factors has not yet been fully established, these findings should be interpreted with appropriate caution and warrant further validation before broader clinical application.

## Data Availability

The datasets presented in this study can be found in online repositories. The names of the repository/repositories and accession number(s) can be found in the article/supplementary material.
